# An efficient self-attention network for skeleton-based action recognition

**DOI:** 10.1038/s41598-022-08157-5

**Published:** 2022-03-08

**Authors:** Xiaofei Qin, Rui Cai, Jiabin Yu, Changxiang He, Xuedian Zhang

**Affiliations:** 1 School of Optical-Electrical and Computer Engineering, University of Shanghai for Science and Technology, Shanghai, 200093 China; 2 Key Laboratory of Industrial Internet and Big Data, China National Light Industry, Beijing, 100048 China; 3 School of Artificial Intelligence, Beijing Technology and Business University, Beijing, 100048 China; 4 College of Science, University of Shanghai for Science and Technology, Shanghai, 200093 China; 5 Shanghai Institute of Intelligent Science and Technology, Tongji University, Shanghai, 200093 China; 6 Shanghai Key Laboratory of Contemporary Optics System, Shanghai, 200093 China; 7 Key Laboratory of Biomedical Optical Technology and Devices of Ministry of Education, Shanghai, 200093 China

**Keywords:** Computer science, Information technology

## Abstract

There has been significant progress in skeleton-based action recognition. Human skeleton can be naturally structured into graph, so graph convolution networks have become the most popular method in this task. Most of these state-of-the-art methods optimized the structure of human skeleton graph to obtain better performance. Based on these advanced algorithms, a simple but strong network is proposed with three major contributions. Firstly, inspired by some adaptive graph convolution networks and non-local blocks, some kinds of self-attention modules are designed to exploit spatial and temporal dependencies and dynamically optimize the graph structure. Secondly, a light but efficient architecture of network is designed for skeleton-based action recognition. Moreover, a trick is proposed to enrich the skeleton data with bones connection information and make obvious improvement to the performance. The method achieves 90.5% accuracy on cross-subjects setting (NTU60), with 0.89M parameters and 0.32 GMACs of computation cost. This work is expected to inspire new ideas for the field.

## Introduction

Human action recognition is an important task that can be used in video analysis, human-computer interaction and so on^1–3^. There are two kinds of human action recognition methods: video-based and skeleton-based. The main challenges of video-based methods are variation of background, viewpoint and people appearance, etc. Some researchers^4^ obtained multi-view videos of the human body through multiple cameras, and fused these videos to cope with these challenges. 3D skeleton data is a type of well-structured data of human major joints and can be regarded as the refinement of video data, which can be easily obtained by using depth camera and pose estimation technology^5^. Compared with video data, skeleton data is more robust to the above challenges^6^.

Skeleton data naturally structured into graph by connecting major points according to nature links in human body structure. Every joint represents node (or vertex), every bone represents edge. That is why many researchers use graph convolution network (GCN) to solve skeleton-based action recognition in recent years. But the nature links of human structure just process one local neighborhood at one graph convolution layer. For example, when people clap hands, the movements of two hands contain most important information. But the features of two hands have to be transferred through two arms and chest, finally fused together. Self-attention mechanism is an effective algorithm to solve such long-distance dependence problems.

Self-attention mechanism has been widely used recently to improve modeling capabilities of GCN in skeleton-based action recognition^7,8^. Some previous approaches have discussed the self-attention mechanism in spatial perspective but without systematically discussing the design approach of the self-attention mechanism in the spatial, temporal, and spatio-temporal perspectives. Inspired by non-local neural network^9^, the self-attention mechanism is discussed from these three perspectives in this work.

Researchers usually use two-stream or multi-stream methods to improve accuracy, but this brings about several times of parameters and computation costs^7,10–13^. These multi-stream methods fuse high-level features of joints and bones at the end of each stream^7,12^. But in another view, low-level features from joints and bones can also be fused together to enrich prior information and generate more representative features. In this work, a trick is used which plays an important role in achieving better performances. The representations of bones and joints are concatenated together at the input layer and use a single-stream network to achieve the same performance of multi-stream network. Unlike those multi-stream methods, this method basically does not increase the calculation costs.

**Figure 1:**
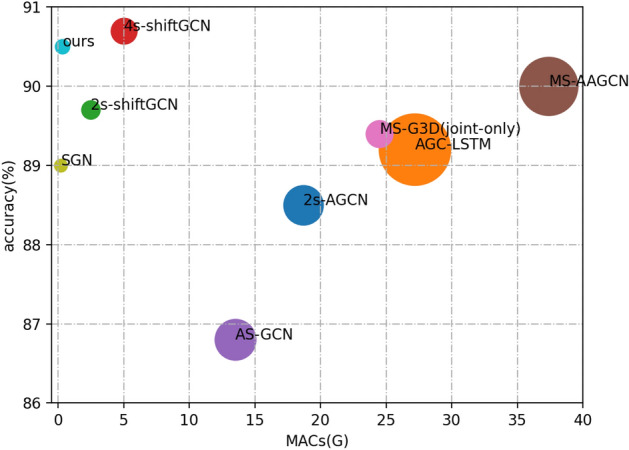
Comparisons of state-of-the-art methods in cross-subject setting (NTU60). The bubble size reflects the parameters of the method , and the center of the bubble represents the computation cost and accuracy.

The contributions of this work are as follows. Firstly, various variants of self-attention network based on a general structure are systematically proposed and discussed for the task of skeleton-based action recognition. Secondly, a trick to enhance the representation capability of skeleton data is proposed, which significantly improves the accuracy while introducing few parameters and computational costs. Finally, based on these innovations, a new network architecture is designed, and the comparison with some state-of-the-art methods is shown in Fig. 1.

## Related work

### Skeleton-based action recognition

The goal of this task is using skeleton data to recognize the action of instance. The input is skeleton sequence in the form of a graph, and what needs to be requested is the class of action. Skeleton data consists of two parts, one part is a vector composed of joint point positions, and another part is a matrix formed by the connection relationship of the joints.

Several years ago, convolution neural networks (CNNs) and random forest (RF) were widely used to deal with the task. But CNNs fail to model the structure of skeleton data properly because skeleton data are naturally embedded in the form of graphs rather than a vector sequence or 2D grids. After firstly applied to this task in ST-GCN^14^, GCNs have been the mainstream methods and make great achievements. AGC-LSTM^15^ proposed another idea on how to use GCNs in this task, and step further to higher accuracy. In these algorithms, the graph of nature links plays a significant role. Some researchers optimize the graph structure by adding edges which is hand-designed, such as MS-G3D^11^. Some other researchers proposed adaptive GCNs^7,12,16^, which produce the dependencies totally different from the graph of human structure. All in all, these methods tried to solve the problem of dependencies in space. In another view, the major joints locations represent the poses in each frame, and the changes of posture determine the action. The dependencies between frames should also be considered. Some methods added links or made a shift in the features between adjacent frames^10,14,17,18^. Some others transferred the module that was often used to process time series, such as recurrent neural network (RNN) and long short-term memory (LSTM), to a new one by replacing CNN units with GCN ones^15,19^. Most recently, some researchers have generated adjacent matrix dynamically by using self-attention mechanism and lower the complexity of networks^7,8^. However, these researchers discussed the self-attention mechanism only in the spatial dimension.

### Graph model

Graph is a kind of data structure which models a set of objects (nodes) and their relationships (edges). Recently, researches of analyzing graphs with machine learning have received more and more attention for its wide applications^20–22^. As a unique non-Euclidean data structure for machine learning, graph analysis focuses on node classification, link prediction, and clustering. Inspired by CNN which is the most popular methods in many fields, GCN is generated. As the input of GCN, the nodes signals are embedded in a vector, whose relationships are embedded in a matrix named adjacent matrix. Graph model can be divided into directed graphs and undirected graphs, and their adjacent matrixes are different. Adjacent matrix is symmetric in undirected graphs, and it is not symmetric in directed graphs.

### Self-attention mechanism

Self-attention mechanism has been successfully used in a variety of tasks. Attention mechanism can be described as $Attention(Query, Source)=\Sigma ^{L_{x}}_{i = 1} Similarity(Query, Key_{i}) \cdot Value_{i}$^23^. When Query, Key, Value are same, it is self-attention mechanism. Non-local neural network is a kind of self-attention application in computer vision.

In brief, self-attention mechanism exploits the correlation in a sequence, and each position is computed as the weighted sum of all positions. The weight of every position in similarity matrix is generated dynamically. The proposed self-attention block is transferred from non-local neural network. It works like an abstract graph neural network and the similarity matrix can be seen as a weighted adjacent matrix. Some researchers have discussed the designs and effects of self-attention mechanism on the task of human skeleton-based action recognition, and used it to model spatial dependencies of the human skeleton. However, in addition to spatial dependencies, temporal and spatio-temporal dependencies can also be modeled by the self-attention mechanism.

## The methods

### Pipeline

**Figure 2:**
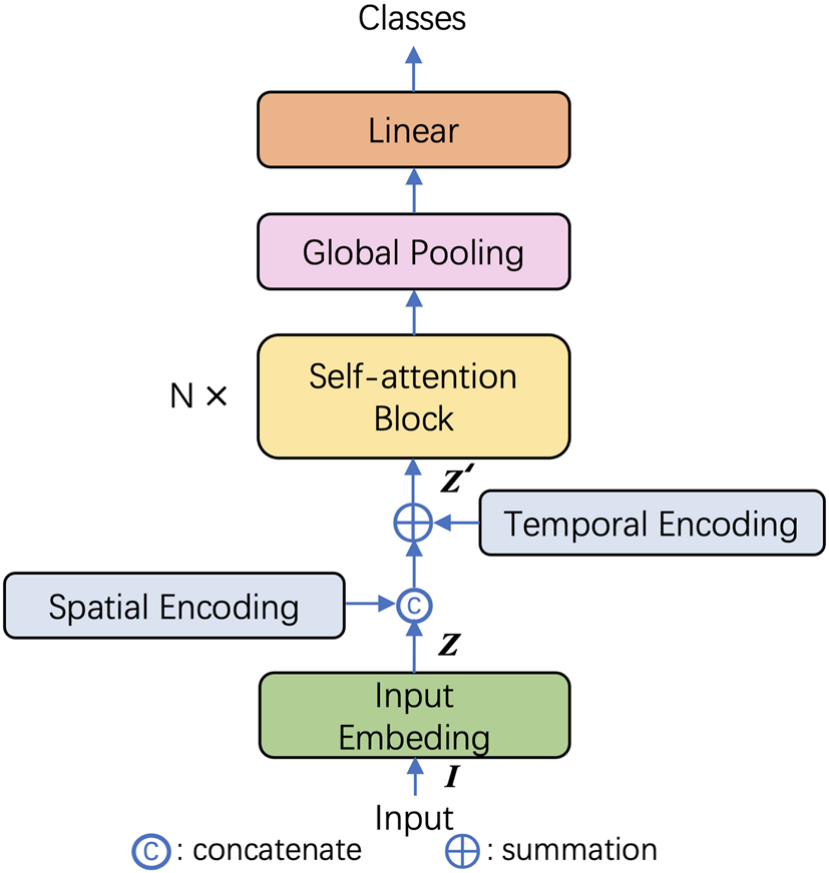
Model architecture.

The framework of the network is shown in Fig. 2. For the original skeleton data of position ${P} \in \mathbb {R}^{C \times V \times T}$, *C* denotes the channel number, *V* and *T* denote the numbers of joints and frames.

**Figure 3:**
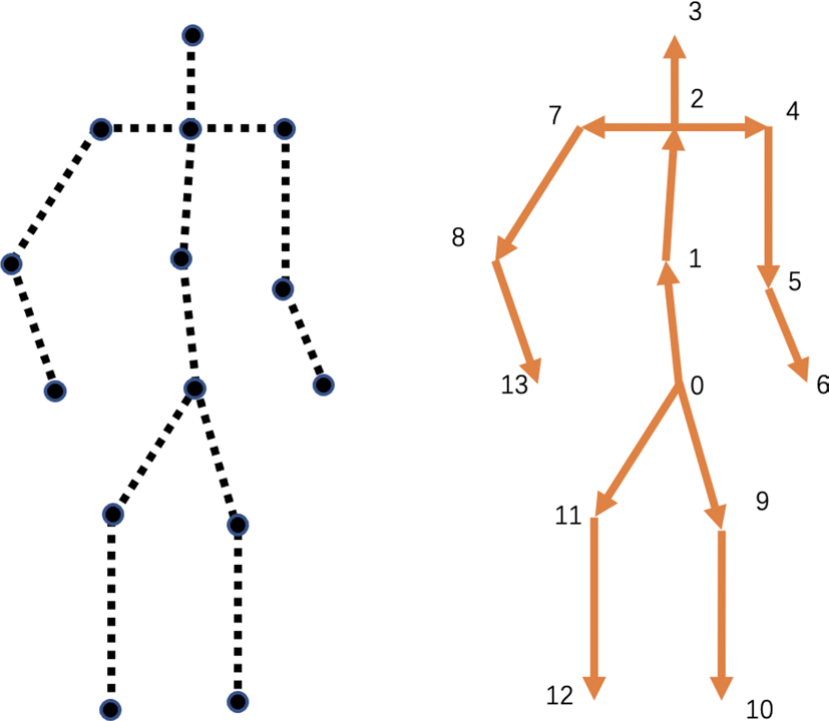
Representations of joints and bones. The left part shows the representation of joints which is naturally embedded into graph. The right shows the representation of bones.

Different from position input, the original input is enriched with bones information and it is named multi-representation method. As shown in Fig. 3, a root joint is set, whose index is 0 in the skeleton data. Every joint is transformed to a vector which points from front joint to the current one, while the vector of root joint is 0. In order to compute easily, firstly an identity matrix ${W}_{J \times J}$ is given, then set some elements whose column index is same to the directed connection joints to be $-1$. For example, there are connected nodes ${p}_{2} , {p}_{1}$, directed edge ${e _{2}}$ is calculated by ${e}_{2} = {p}_{2} - {p}_{1} = ((x_{2} - x_{1}),(y_{2} - y_{1}),(z_{2} - z_{1}))^{T}$ and the element (2,1) in ${W}$ is set to be $-1$. The representation of bones is ${E} = {P} \cdot {W}$. Then ${E}$ and ${P}$ are concatenated as the input of the network:

1 $$\begin{aligned} {I} = cat({P}, {P}\cdot {W}) \end{aligned}$$

where $${I}\in \mathbb {R}^{2C \times V \times T}$$.

Firstly, the velocities of human joints and bones are calculated separately. It is easy to understand that the movements of joints are important for the action recognition and it is calculated as:

2 $$\begin{aligned} {v}_{p}^ {t+1} = {p}^{t+1} - {p}^{t} \end{aligned}$$

Especially, $${v}_{p}^ {t=0} = 0$$.

But some movement of the joints may caused by the front joints movement which is absolute motion that sometimes should be ignored. For example, when reaching for something, there is a considerable movement of the hand, which is actually caused by the movement of the elbow. In this case, the actual grasp movement of the hand is ignored because the grasp movement of the hand is not obvious compared to this large movement of the elbow. And the difference between adjacent frames of the vectors presenting bones between is relative motion:

3 $$\begin{aligned} {v}_{{e}_{n}}^ {t+1} = {e}_{n}^{t+1} - {e}_{n}^{t} =({p}_{n}^{t+1} - {p}_{n-1}^{t+1}) - ({p}_{n}^{t} - {p}_{n-1}^{t}) = ({p}_{n}^{t+1} - {p}_{n}^{t}) - ({p}_{n-1}^{t+1} - {p}_{n-1}^{t}) \end{aligned}$$

Similarly, $${v}_{{e}_{n}}^ {t=0} = 0$$.

In every moment, the ${v}^{t}$ can be calculated, then they can be concatenated to have ${V}$ and ${V}\in \mathbb {R}^{2C \times V \times T}$. Then the ${V}$ is embeded into high dimensional space by two $1 \times 1$ convolution layers:

4 $$\begin{aligned} {\tilde{V}} = ReLu({W}_{4}(ReLu({W}_{3}{V} ))) \end{aligned}$$

Similarly, the ${I}$ is embeded into same high dimensional space:

5 $$\begin{aligned} {\tilde{I}} = ReLu({W}_{2}(ReLu({W}_{1}{I} ))) \end{aligned}$$

And they are fused together by summation:

6 $$\begin{aligned} {Z} = {\tilde{V}} + {\tilde{I}} \end{aligned}$$

where ${W}_{1}, {W}_{3}\in \mathbb {R}^{C_{1}\times 2C}$ and ${W}_{2}, {W}_{4}\in \mathbb {R}^{C_{1}\times C_{1}}$, *ReLu* denotes the ReLu activation function.

After embedding the input signals, ${Z}$ is fused with the encoded joint type and frame index. One-hot encoding is adapted to encode the semantics of joint type and frame index, then the method described in Eqs. (4) and (5) is used to promote representativity of semantics by mapping them into higher dimension. Finally, fuse the encoded semantics of time and space with ${Z}$:

7 $$\begin{aligned} {Z'} = cat({Z}, {\tilde{J}}) + {\tilde{T}} \end{aligned}$$

${\tilde{J}}$ and ${\tilde{T}}$ is the encoded semantics of joint type and frame index.

After several stacks of self-attention blocks which will be illustrated in next section, the feature maps are pooled from $\mathbb {R}^{C \times T \times V}$ to $\mathbb {R}^{C \times 1 \times 1}$ in global pooling layer. Finally, after a linear layer, the classes of the actions are generated.

### Self-attention block

**Figure 4:**
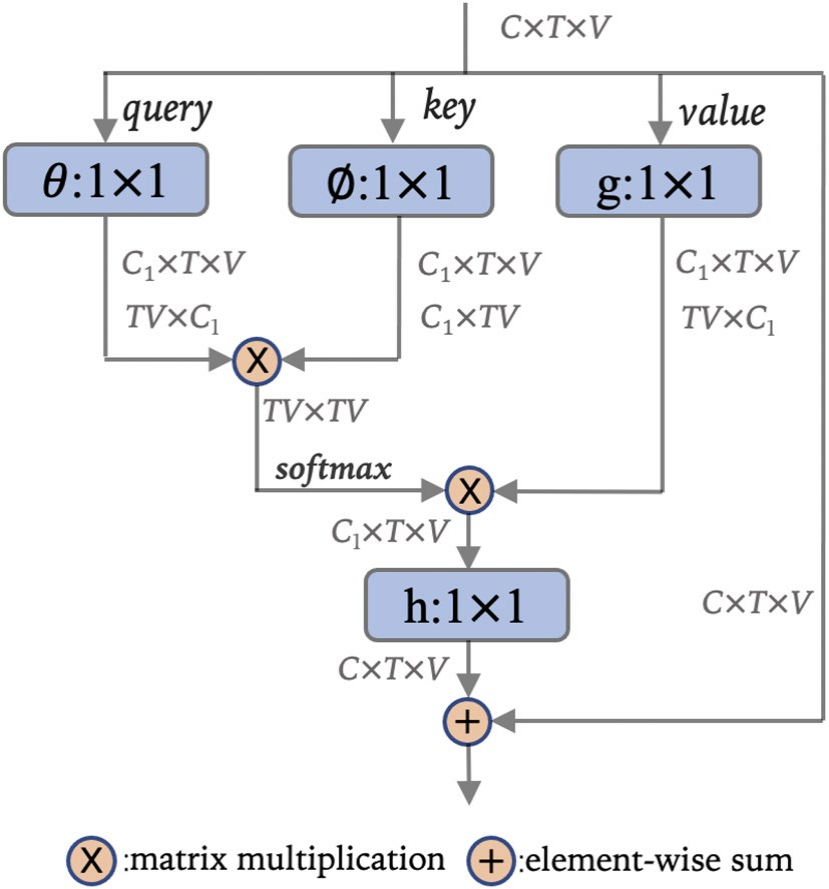
Spatio-temporal self-attention block. C is the channel size; T and V are the number of frames and joints. $\theta$, $\phi$, g and h denote $1 \times 1$ convolution. If $C_{1} < C$, it is a kind of bottleneck.

Figure 4 shows a spatio-temporal self-attention block. Some reshaping operations are designed that vary from the types of self-attention block.

${x}$ denotes input signal and ${y}$ denotes output in the following illustration of the algorithm. And the general self-attention block can be described as:

8 $$\begin{aligned} {y}({x}) = ReLu({h} ({f}({x}) \cdot {g}({x}))+ {x}) \end{aligned}$$

${f}({x})$ is a function to generate the similarity matrix: ${f}({x}) = softmax(\theta ({x})^{T}\phi ({x}))$. And ${g}({x})$ and ${h}({x})$ are linear embedding: ${g}({x}) = {W}_{{g}}{x}$, ${h}({x}) = {W}_{{h}}{x}$. Actually it is a $1 \times 1$ convolution operation. The final $+ {x}$ denote a residual connection.

Same to ${g}({x})$, $1 \times 1$ convolution operation is used to embed the Query and Key: $\theta ({x}) = {W}_{\theta }{x}$, $\phi ({x}) = {W}_{\phi }{x}$. And the ${f}({x})$ can be calculated as:

9 $$\begin{aligned} {f}({x}) = softmax(({W}_{\theta }{x})^{T}({W}_{\phi }{x})) \end{aligned}$$

The ${W}_{{h}}, {W}_{\theta }, {W}_{\phi }, {W}_{{g}}$ are learnable.

The reshaping operation that is not described in the equations is illustrated in Table 1. In spatio-temporal self-attention block, the feature maps of $\theta$ operation is reshaped to $\mathbb {R}^{TV \times C_{1}}$, and we denote this as $\theta ({x})^{T} \in \mathbb {R}^{TV \times C_{1}}$. Similarly, $\phi ({x}) \in \mathbb {R}^{C_{1} \times TV }$, ${g}({x}) \in \mathbb {R}^{TV \times C_{1}}$.

**Table 1:** Reshaping operations.

	$\theta \; ({x})^{T}$	$\phi \; ({x})$	${f}\;({x})$
*ST*	$TV \times C_{1}$	$C_{1} \times TV$	$TV \times TV$
$S_{1}$	$T \times V \times C_{1}$	$T \times C_{1} \times V$	$T \times V \times V$
$S_{2}$	$V \times TC_{1}$	$TC_{1} \times V$	$V \times V$
$T_{1}$	$V \times T \times C_{1}$	$V \times C_{1} \times T$	$V \times T \times T$
$T_{2}$	$T \times VC_{1}$	$VC_{1} \times T$	$T \times T$

The types of self-attention blocks vary from the different reshaping operation.

*ST*: spatio-temporal block; $S_{1}$ and $S_{2}$: two kinds of spatial blocks; $T_{1}$ and $T_{2}$: two kinds of temporal blocks.

In spatial self-attention blocks, the reshaping operations are different. Depending on two kinds of ways dealing with time dimension, there are two kinds of spatial blocks. If the similarity matrix differs in every moment, then $\theta ({x})^{T} \in \mathbb {R}^{T \times V \times C_{1}}$, $\phi ({x}) \in \mathbb {R}^{T \times C_{1} \times V }$, ${g}({x}) \in \mathbb {R}^{T \times V \times C_{1}}$. The similarity matrix is ${f}({x}) \in \mathbb {R}^{T \times V \times V }$ and ${f}({x}){g}({x}) \in \mathbb {R}^{T \times V \times C }$. Another way is that the similarity matrix do not change in every moment, then $\theta ({x})^{T} \in \mathbb {R}^{V \times TC_{1}}$, $\phi ({x}) \in \mathbb {R}^{TC_{1} \times V }$, ${g}({x}) \in \mathbb {R}^{V \times TC_{1}}$. The block is much more like spatio-temporal self-attention block, and it is easy to have ${f}({x}) \in \mathbb {R}^{V \times V }$, ${f}({x}){g}({x}) \in \mathbb {R}^{V \times TC }$.

As for temporal self-attention blocks, there are two kinds of temporal blocks because of the same reason shown in spatial self-attention blocks, these two can be easily obtained by switching *T* and *V* in spatial self-attention block. So there is no need to go into details.

There are some more interesting things to consider. The self-attention block works like dynamical GCN. Actually, the similarity matrix is considered as adjacent matrix, the graph is directed. For example, in one frame, the weight from neck to head may not be same as the weight from head to neck. The positions of these two weights in the similarity matrix are symmetric. Are these two weights same? And should these two be same? Some experiments have been done about these illustrated in experiments section. If the similarity matrix should be symmetric, ${W}_{\phi }$ is set same to ${W}_{\theta }$:

10 $$\begin{aligned} {f}({x}) = softmax(({W}_{\theta }{x})^{T}({W}_{\theta }{x})) \end{aligned}$$

Another problem is how to model the temporal sequences if spatial self-attention block is adapted. Similarly, how to model space if only temporal self-attention block is used? In residual connection, when the channel size of input and output are different, $1 \times 1$ convolution operation is adopted. The $1 \times 1$ convolution operation is replaced in residual connection with $1 \times 3$ or $3 \times 1$ convolution operation to model time or space. The changes of residual connection may not decrease the performance of the network. Because the network is light and there is no need to consider much about learning abilities of the network when stacking deeper.

**Figure 5:**
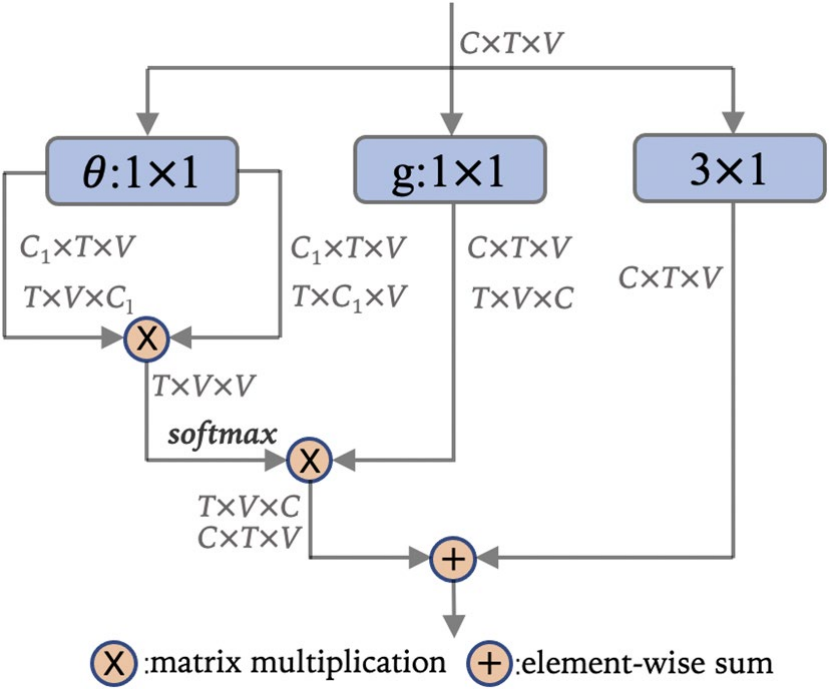
A kind of spatial self-attention block. C is the channel size; T is frame; V is the human joint. $\theta$ and ${g}$ denote $1 \times 1$ convolution. $3 \times 1$ denote $3 \times 1$ convolution.

The generic self-attention operation is flexible. Some convolution operation can be removed from self-attention block in practice. And based on the analysis above, there are many variants of self-attention block. For example, Fig. 5 shows a kind of spatial self-attention block. T is treated as batch size in this spatial self-attention block , and similarity matrix is calculated by Eq. (10).

## Experiments

### Dataset

#### NTU-RGBD^5^

It is a large-scale action recognition dataset containing 56,880 skeleton sequences of 60 action classes, which is also known as NTU60. This dataset is performed by 40 distinct subjects and captured by three Kinect cameras at the same height but from different horizontal angles: $-45^{\circ }$, $0^{\circ }$, $45^{\circ }$. Each human skeleton is represented by 25 joints with 3D coordinates. For Cross-View (CV) settings, skeleton data from two cameras are used for training while the other is used for testing. For Cross-Subject (CS) settings, 40,320 clips from 20 subjects are used for training, and the rest for testing. 10% of the training sequences is randomly selected for validation for both the CS and CV settings.

#### NTU-RGBD120^24^

It is an extension of NTU-RGBD dataset. It contains 114,480 skeleton sequences of 120 action classes performed by 106 distinct subjects. For Cross-Subject settings, half of subjects are used for training while the others for testing. In the Cross-Setup setting, clips of half setup IDs are used for training and the rest for testing.

#### Kinetics skeleton 400^14^

It is a large-scale action recognition dataset containing 240,000 training and 20,000 testing skeleton sequences over 400 classes. The Kinetics 400 dataset is from the Kinetics 400 video dataset^25^ and OpenPose^26^ pose estimation toolbox. Each skeleton graph contains 18 major joints and each joint is represented with a tuple of (X, Y, C), in which the (X, Y) is 2D coordinates in pixel coordinate system and C is the confident scores given by toolbox. For the multi-people cases, two persons with the highest average joint confident scores are selected in each clip.

### Implementation details

#### Data processing

Same to SGN^8^, sequence level translation based on the first frame is performed to be invariant to the initial positions. If one frame contains two persons, this one is split into two frames by making each one contains one skeleton. During training, each skeleton sequence is segmented into 20 clips equally, and randomly select one frame from each clip to get 20 frames. During testing, similar to Glimpse Clouds^27^, 5 sequences are randomly created in the similar manner and final main score is used to predict the class. For data augmentation, the skeleton data is rotated to some degrees. Three angles are randomly generated between [$-17^{\circ }$, $17^{\circ }$] as the rotation angles of X, Y, Z axes for one sequence. Specially, angles is selected between [$-30^{\circ }$, $30^{\circ }$] in NTU-RGBD CV setting, for its large view variation. As for Kinetics 400, two people with highest average joints confidence are selected, and data augmentation is not applied. During test, different from NTU datasets, only one sequence is used.

#### Training details

All of the works are implemented on one GTX 1080ti GPU. Adam optimizer is adopted and the initial learning rate is set to 0.001. The network is trained for 120 epochs, and the learning rate is decayed at 60th, 90th and 110th epoch by a factor of 10. The weight decay is set to be 0.0001. The batch size is set to be 64 for every dataset. Label smoothing loss function is used and the smoothing factor is set to be 0.1.

### Ablation study

In this part, the influences of these self-attention blocks and the multi-representation method are studied on NTU60 dataset. Most comparative experiments are accomplished based on spatio-temporal self-attention block shown in Fig. 4, except the comparisons between every kinds of self-attention block. The self-attention block is stacked four times.

The influence of different representations is shown in Table 2. Compared with two-stream method^7^, the proposed method reached same accuracy with half the amount of parameters. At the beginning of the network, the channel size is small, that is why the method of combining joints and bones at the beginning does not increase the parameters. Actually, the method increases only 0.002 M parameters which can be ignored. But multi-stream method repeats the network and fuse all the features in the final linear layer, that brings much more parameters. Multi-stream method is effective to achieve better results, but it is not cost-effective to increase the amount of calculation exponentially in order to improve a paltry effect.

**Table 2:** Comparison between multi-representation and multi-stream.

	params (M)	cs (%)	cv (%)
J	0.89	89.0	95.1
B	0.89	87.4	94.9
2-stream	1.78	90.6	96.0
J + B (proposed)	0.89	90.5	96.1

J denotes joints only, B denotes bones only, J + B denotes the multi-representation method, 2-stream denotes two-stream method

Table 3 shows the influence of whether the graph is directed. The spatial self-attention block shown in Fig. 5 is used to accomplish this experiments. There is not much difference in accuracy between them, but the algorithm of directed graph has more parameters because one more $1 \times 1$ convolution branch is needed. In this work, undirected graph is used.

The influence of the number of stacked self-attention blocks is shown in Table 4. When the block is stacked deeper, the performance become better. But the performance stops benefiting from more stacked blocks when N is more than 4, and the model starts overfitting to the training set. In this work, N is set to 4.

**Table 3:** Direct graph *vs* undirected graph.

	params (M)	cs (%)	cv (%)
Directed	1.00	90.4	96.1
Undirected	0.89	90.5	96.1

**Table 4:** Comparison between deferent number of stacked self-attention blocks.

N	cs (%)	cv (%)
2	73.0	80.0
3	86.5	90.3
4	90.6	96.1
5	90.5	96.1

Table 5 shows the comparison between different self-attention blocks. As shown in the table, the best results are achieved based on the variant of spatial self-attention block shown in Fig. 5. It is worth noting that the variant of spatial self-attention block has the familiar results with spatio-temporal self-attention block. This shows that the dependencies between joints are much more complex and important than frames. Actually, in the variant of spatial self-attention block, a simple $3 \times 1$ convolution operation is adopted to model the dependencies between frames. In this work, the block shown in Fig. 5 is used.

**Table 5:** Comparisons between different self-attention blocks.

	cs (%)	cv (%)
$S_{1}$	89.6	94.8
$S_{2}$	89.3	94.8
$T_{1}$	87.3	93.4
$T_{2}$	86.5	93.0
*ST*	90.5	96.0
*V*	90.5	96.1

*ST*: spatio-temporal block; $S_{1}$ and $S_{2}$: two kinds of spatial blocks; $T_{1}$ and $T_{2}$: two kinds of temporal blocks. Their definitions are shown in Table 1. *V* denotes the block that shown in Fig. 5.

### Comparisons with other methods

This work concentrates on both accuracy and computation costs. The final model is compared with many state-of-the-art skeleton-based action recognition methods. In Table 6, the accuracy and the amount of parameters are compared with many influential methods on NTU60. The values of parameters amount in some paper are not given, in this case, they are gotten by using ptfloaps and their source code. If their source code is not available, the value of parameters in Table 6 is indicated by a “–”. 5 sequences are created and the final main score is used for testing while one sequence for training, so the testing computation cost is 5 times of training. In addition, Fig. 1 make comparisons more intuitively. The results show that the size of the network is much smaller than most other methods. Comparisons on NTU120 and Kinetics400 are shown in Tables 7 and 8.

**Table 6:** Performance comparisons on NTU60 with the CS and CV settings in top-1 accuracy.

Methods	Parameters (M)	cs (%)	cv (%)
GCA-LSTM^28^	–	74.4	82.8
VA-LSTM^29^	–	79.2	87.7
ST-GCN^14^	–	81.5	88.3
DPRL+GCNN^30^	–	83.5	89.8
SR-TSL^31^	–	84.8	92.4
AS-GCN^18^	7.40	86.8	94.2
GR-GCN^17^	–	84.8	92.4
2s-AGCN^7^	6.92	88.5	95.1
AGC-LSTM^15^	22.81	89.2	95.0
2s-SDGCN^13^	–	89.6	95.7
SGN^8^	0.69	89.0	94.5
DGNN^32^	8.16	89.9	96.1
Shift-GCN (2s)^10^	1.48	89.7	96.0
Shift-GCN (4s)^10^	2.94	90.7	96.5
MS-G3D (joint)^11^	3.20	89.4	95.0
MS-G3D (2s)^11^	6.40	91.5	96.2
MST (joint)^33^	3.0	89.0	95.1
MST (2s)^33^	6.0	91.1	96.4
Double-head (joint)^34^	3.0	90.3	96.1
Double-head (2s)^34^	6.0	91.7	96.5
Ours	0.89	90.5	96.1

**Table 7:** Performance comparisons on NTU120 with the C-subjects and C-settings in top-1 accuracy.

Methods	Year	c-sub (%)	c-set (%)
ST-LSTM^35^	2016	55.7	57.9
GCA-LSTM^28^	2017	58.3	59.2
Pose Evolution Map^36^	2018	64.6	66.9
2s-AGCN^7^	2019	82.5	84.9
Shift-GCN^10^	2020	85.9	87.6
MS-G3D^11^	2020	86.9	88.4
SGN^8^	2020	79.2	81.5
MST (joint)^33^	2021	82.8	84.5
MST (2s)^33^	2021	87.0	88.3
Double-head (joint)^34^	2021	84.6	85.9
Double-head (2s)^34^	2021	87.9	89.1
Ours	–	85.7	86.8

**Table 8:** Performance comparisons on Kinetics400 dataset.

Methods	Year	Top-1 (%)	Top-5 (%)
ST-GCN^14^	2018	30.7	52.8
AS-GCN^18^	2019	34.8	56.5
2s-AGCN^7^	2019	36.1	58.7
DGNN^32^	2019	36.9	59.6
MS-AAGCN^12^	2019	37.8	61.0
MS-G3D^11^	2020	38.0	60.9
MST (2s)^33^	2021	37.8	60.3
Double-head (joint)^34^	2021	36.6	59.5
Double-head (2s)^34^	2021	38.3	61.1
Ours	–	37.6	60.1

The method achieves competitive accuracy with few parameters and little computation cost. Surely, more attention should be paid to the comparisons with SGN^8^, because the size of this network is similar to the proposed method. Compared to SGN, the accuracy is increased about 1.5% in cross-subjects setting (NTU60) at the cost of 0.2M extra parameters. In NTU120, the accuracy is increased about 6.5% in cross-settings and 5.3% in cross-subjects. As shown in Tables 7 and 8, the methods do not have very impressive accuracy. This is due to the complexity of the dataset. NTU120 and Kinetics400 are very complicated but the network is too lightweight that it may not be able to model the data properly.

### Complexity discussion

The proposed network is very lightweight with 0.89M parameters and 0.32GMACs of computation cost. The following technologies are the key reasons that make the network so lightweight.

Firstly, every sequence is processed to only 20 frames. Most previous methods are based on ST-GCN^14,37^ and every sequence contains 150 frames. The size of data is much larger than the proposed method. More data comes with more information, but also more noise. With 150 frames, the networks have to be stacked deeper to obtain larger temporal receptive field. But in the proposed method, with 20 frames, fewer CNN layers are enough to model the time. Moreover, the motion of every joints and bones is computed which also contains some information about time. This allows us to model time with ease.

Secondly, different from GCN, the self-attention block has global receptive field, there is no need to stack the blocks deep to obtain enough receptive field. So the proposed method based on self-attention mechanism could exploiting the long-range dependencies better with fewer stacked layers.

Thirdly, most operations in the proposed method are linear operations achieved by $1\times 1$ convolution. The parameters amount of $1\times 1$ convolution can be calculated as the input channel number times the output channel number. This operation requires little memory for parameters. The proposed self-attention block is based on non-local neural network. Although non-local neural network is computationally intensive when the input has high resolution, when it comes to skeleton-based action recognition, the input can be regarded as low resolution image about $25\times 20$, which reduces the computation cost much.

Finally, the channel size is not set to be very large, which makes the network lighter. But this also brings us some problems. NTU120 and Kinetics400 are large-scale datasets with hundreds of action classes, which requires more feature channels to represent them. The proposed network is too lightweight to model such complex data, and do not achieve very impressive performance on these two datasets.

## Conclusion

In this work, the application of self-attention mechanism in the task of skeleton-based action recognition is systematically analyzed and discussed, and a variety of self-attention modules are designed, which can be regarded as different adaptive graph convolution modules. Based on these modules, a novel model architecture is proposed. In addition, the trick of using low-level feature fusion instead of high-level feature fusion is proposed to improve network efficiency without bringing in additional computation costs and parameters. The method overperforms most previous methods in accuracy on NTU60 dataset. For those methods with higher accuracy, the parameters and calculations of the proposed method are dozens of times smaller than them. The proposed method in this paper may inspire the research of graph models in other fields. We will also further investigate the application of self-attention graph models in other tasks.
